# Rare case of fulminant myocarditis in a patient with rickettsia infection

**DOI:** 10.21542/gcsp.2026.15

**Published:** 2026-04-30

**Authors:** Nikolaos Tsiamis, Dimitrios Afendoulis, Christos Tountas, Fotis Toulgaridis, Flora Tsakirian, Eleftherios Vidalakis, Georgios Totikidis, Konstantinos Toutouzas, Anastasia Kitsiou

**Affiliations:** 1 Sismanogleio General Hospital, Cardiology Department, Athens, Greece; 2 Unit of Structural and Valvular Heart Diseases, 1 st Department of Cardiology, NKUA, ‘Hippokration’ General Hospital of Athens, Greece

## Abstract

We report a rare case of fulminant myocarditis caused by *Rickettsia conorii* infection in a 31-year-old previously healthy male from rural Greece. The patient presented with acute chest pain, fever, and an insect bite eschar. Initial electrocardiography (ECG) demonstrated inferior ST-segment elevation, and coronary angiography excluded obstructive coronary artery disease. Within 24 h, the patient deteriorated to hemodynamic collapse, with left ventricular ejection fraction (LVEF) declining to 20–25%. Management included doxycycline, empirical broad-spectrum antibiotics, intravenous corticosteroids, and aggressive diuresis. Serological confirmation of *R. conorii* infection (IgM titer 1:64) directed targeted antibiotic monotherapy. Cardiac magnetic resonance (CMR) imaging performed two days post-discharge confirmed acute myocarditis per the 2018 Updated Lake Louise Criteria, with extensive late gadolinium enhancement (LGE) involving 28% of LV mass. LVEF recovered to 56% within weeks. This case highlights the potential for *R. conorii* to precipitate fulminant myocarditis even in immunocompetent young patients, underscoring the importance of clinical suspicion in endemic regions.

## Case presentation

**Figure 1.**
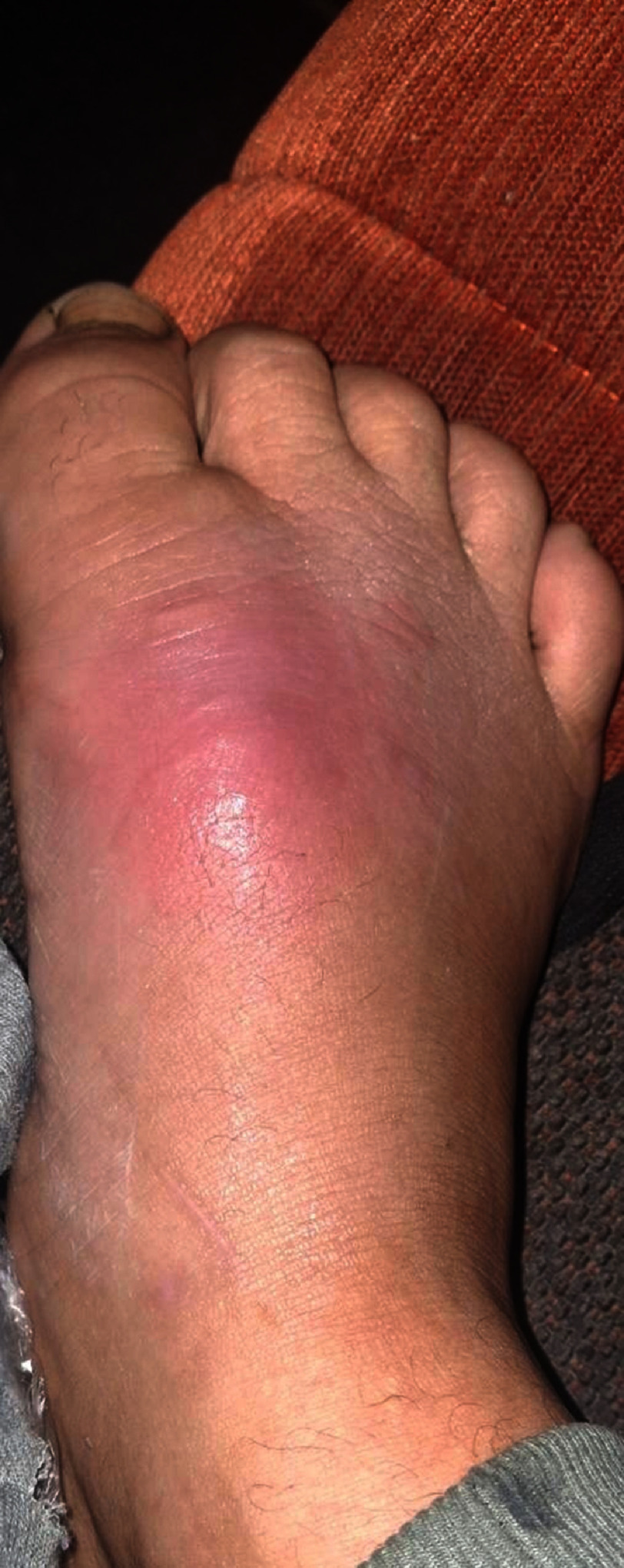
Insect bite on the dorsal surface of the right leg.

**Figure 2.**
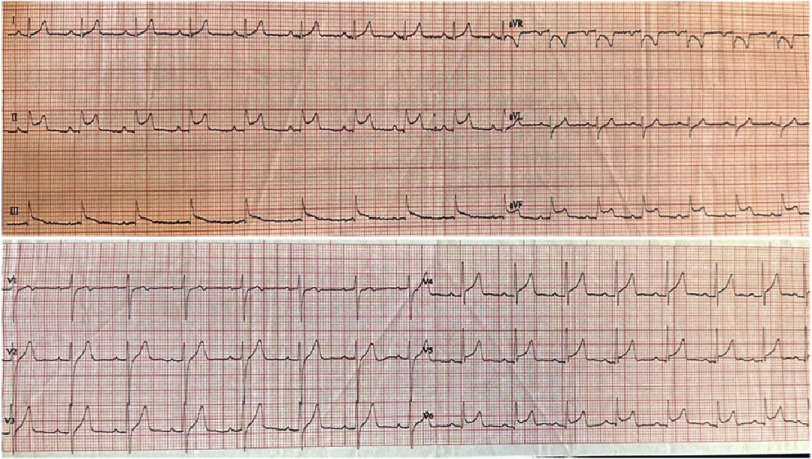
Initial ECG depicted sinus rhythm with ST elevation in leads II, III, aVF, V5 and V6, and ST-segment depression in aVR. Heart rate; 80 bpm.

A 31-year-old male from a rural area presented to the emergency department with acute-onset chest pain accompanied by weakness and fatigue of 9 hours’ duration. He reported a 4-day history of fever without chills, in association with an insect bite on the dorsal surface of the right leg showing signs of local inflammation (Figure 1). Initial ECG demonstrated sinus rhythm with ST-segment elevation in leads II, III, aVF, V5, and V6, and ST-segment depression in aVR, at a heart rate of 80 bpm (Figure 2). Transthoracic echocardiography (TTE) on presentation revealed a preserved LVEF and normal LV dimensions, with mild hypokinesia of the basal inferoseptal and basal-to-mid inferolateral walls. No valvulopathy or pericardial effusion was identified.

### Further diagnostic evaluation and management

Coronary angiography demonstrated unobstructed epicardial coronary arteries. The patient was admitted to the Cardiology Intensive Care Unit for further investigation and management. Laboratory results revealed elevations in C-reactive protein (CRP), white blood cell count, and liver enzyme levels, as well as a markedly elevated high-sensitivity troponin-I at 18,722 pg/mL (Table 1). Blood and urine cultures were obtained. Serological testing was initiated for IgM antibodies against Coxsackievirus, Epstein–Barr virus (EBV), cytomegalovirus (CMV), *Toxoplasma gondii*, parvovirus B19, and *Rickettsia conorii*; additional testing for *Rickettsia*, *Borrelia*, and Echovirus was sent to the Microbiological Laboratory of the Medical School, University of Athens. An autoimmune panel—comprising rheumatoid factor (RF), antinuclear antibodies (ANA), antimitochondrial antibodies (AMA), c-ANCA, p-ANCA, C3, and C4—was also obtained (Table 1). Testing for *Mycoplasma pneumoniae* was negative.

**Table 1:** The laboratory work-up.

**Parameter**	**Normal Values**	**On Admission**	**Day 1**	**Day 12**	**Follow Up 1 Month**
WBC	4,000–11,000/µL	13,690	13,680	6,500	6,000
NEU	2,000-8,000/µL	8,800	11,270	4,770	4,600
LYM	1,100–4,000/µL	2,860	1,280	1,500	1,250
MONO	100–900/µL	1,910	1,100	200	150
EOS	100–500/µL	90	10	20	10
BASO	0.0–200/µL	30	20	10	0
RBC	4.5–5.9 × 10^3^/µL	5.18	4.76	5.7	5.5
HGB	13.5–17.5 g/dL	15.2	14	15.2	16
HCT	41.0–53.0%	44.90%	40.20%	46%	48.20%
MCV	76.0–96.0 fL	83.8	84.5	84	84
MCH	27.0–33.0 pg	28.4	29.4	29	28.6
Quick PT	10.00–13.00 s	13.8	13.7	11.5	
INR	0.86–1.20	1.202	1.2	1.05	
APTT	24.0–39.0 s	32.56	34.1	31	
Fibrinogen	1.80–4.00 g/L	5.17	4.5	3.6	
D-dimer	<500.0 µg/L	360.76	450	300	
PLT	150,000–400,000/µL	339,000	228,000	250,000	280,000
ESR	0–15 mm/h	30		10	
Urea	10–50 mg/dL	44	50	30	20
Creatinine	0.5–1.5 mg/dL	0.8	0.7	0.8	0.7
Total Bilirubin	0.20–1.40 mg/dL	0.68	1.09	1.1	1
Direct Bilirubin	0.00–0.50 mg/dL	0.3	0.49	0.5	0.42
ALP	42–128 U/L	73	90	45	49
ALT	4–45 U/L	17	90	35	18
AST	4–45 U/L	66	251	30	21
LDH	135–225 U/L	650	943	160	140
CPK	25–190 U/L	622	1547	150	40
Na	135–148 mEq/L	139	139	137	141
K	3.5–5.3 mEq/L	3.7	4.4	4.7	4.6
hsTnI	38–80 pg/mL	13,734	18,722	120	<2
CRP	<6 mg/L	85.8	140.6	10	<2
BNP	<100 pg/ml	597	645	105	39
PCT	<0.5	0.1		0.1	
Tryptase	<11.4 µg/L		4.2		
TSH	0.27–4.20 mIU/L		0.25		
FT4	0.8–2.0 ng/dL		1.6		
Anti-Rickettsia rickettsii IgG		Negative (<1:64)			
Anti-Rickettsia rickettsia IgM		Negative (<1:64)			
Anti-Rickettsia typhi IgG		Negative (<1:64)			
Anti-Rickettsia typhi IgM		Negative (<1:64)			
Anti-Rickettsia conorii IgG		Negative (<1:64)			
Anti-Rickettsia conorii IgM		**Positive (>1:64)**			
Anti-Borrelia IgG		Negative (10.6 U/ml)			
Anti-Borrelia IgM		Negative (11.5 U/ml)			
Mycoplasma Pneumoniae IgM	<9 U/ml	Negative (5 U/ml)			
Anti-Coxsackie IgM	<0.9 S/CO	Negative (0.27 S/CO)			
Anti-ECHO IgM	<10 U/ml	Negative (5.4 U/ml)			
TOXO-IgM	<0.5 index	Negative 0.09 index			
CMV-IgM	<0.85 S/CO	Negative 0.21 S/CO			
EBV-IgM	<0.5 U/ml	Negative 0.05 U/ml			
IgG	859–1517 mg/dL	1210			
IgM	64.5–195 mg/dL	160			
IgA	127–348 mg/dL	189			
IgE	<165 IU/mL	279			
C3	88–135 mg/dL	165			
C4	16–27 mg/dL	58.3			
RF	<20 IU/ml	<16 IU/mL			
ANA	<1:160	Negative			
AMA	<1:160	Negative			
ASMA	<1:160	Negative			
c-ANCA	<1:20	Negative			
p-ANCA	<1:20	Negative			
Anti-PR3	<10 U/ml	Negative (1.4 U/ml)			
Anti-MPO	<5 U/ml	Negative (0.4 U/ml)			
Anti-HAV IgM	<0.8 S/CO	Negative (0.31 S/CO)			
HbsAg	<1 S/CO	Negative (0.47 S/CO)			
Anti-HBc IgM	<1 S/CO	Negative (0.12 S/CO)			
Anti-HCV	<1 S/CO	Negative (0.1 S/CO)			
HIV I/II	<1 S/CO	Negative (0.07 S/CO)			
Parvovirus B19	<0.9 index	Negative (0.1 index)			
PCR Covid 19		Not detected			
PCR Influenza		Not detected			
PCR RSV		Not detected			

**Figure 3.**
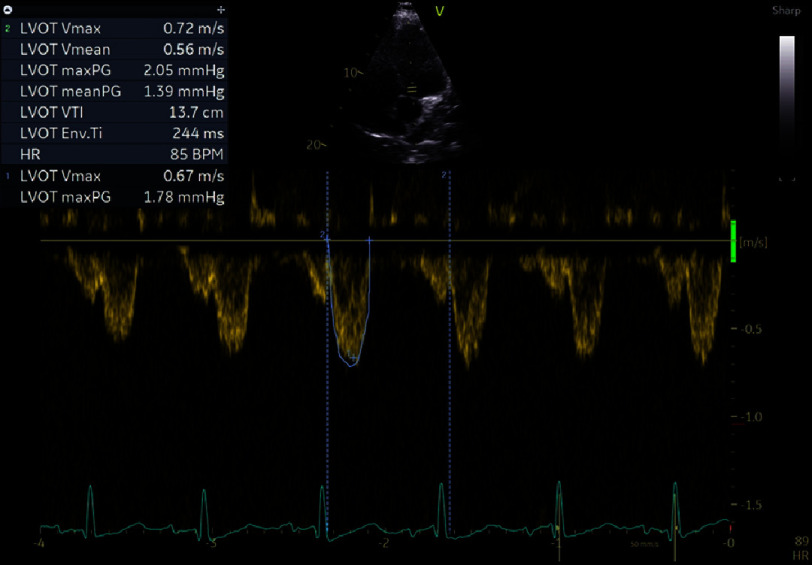
Initial echocardiography depicting reduced VTI in LVOTO, consistent with low cardiac output.

On day 1 of hospitalization, the patient’s clinical status deteriorated rapidly, with hemodynamic collapse. Bedside TTE revealed a severely reduced LVEF (estimated 20–25%), diffuse LV hypokinesia, and signs of markedly reduced cardiac output (LVOT VTI: 13.7 cm) (Figure 3). The right ventricle (RV) was dilated with impaired systolic function, mild-to-moderate mitral regurgitation had developed, and the inferior vena cava (IVC) was dilated without respiratory variation, consistent with elevated right atrial pressure. The pericardium remained free of effusion.

Despite the severe reduction in contractility and evidence of systemic congestion, the patient remained hemodynamically stable without requiring inotropic agents or mechanical circulatory support. Management was directed toward achieving a strictly negative fluid balance with intravenous diuretic therapy and supplemental oxygen. Serial arterial blood gas analyses demonstrated a progressive decline in lactate levels, reflecting adequate systemic perfusion despite the severely reduced LVEF. Over the following days, oxygen requirements decreased in parallel with clinical and echocardiographic improvement.

Following an infectious disease consultation, intravenous ampicillin/sulbactam was added to the existing doxycycline regimen as empirical broad-spectrum coverage, given the patient’s acute febrile presentation and markedly elevated inflammatory markers, pending definitive serological results. Concurrently, intravenous methylprednisolone pulses were initiated as a rescue strategy for suspected acute myocarditis, in accordance with the 2025 ESC Guidelines for the Management of Myocarditis and Pericarditis, which assign a Class IIb recommendation to corticosteroid use in acute myocarditis complicated by impaired LV function or hemodynamic instability refractory to standard heart failure therapy^1^. A quaternary center for advanced heart failure was alerted to prepare for the potential need for mechanical circulatory support and endomyocardial biopsy (EMB). Following the initial corticosteroid pulses and supportive care, the patient demonstrated prompt clinical and hemodynamic improvement; no further immunosuppressive doses were administered, and transfer for EMB was ultimately deemed unnecessary.

Following clinical stabilization, guideline-directed medical therapy (GDMT) for heart failure was initiated, comprising low-dose ACE inhibitor, beta-blocker, and mineralocorticoid receptor antagonist (MRA), gradually uptitrated as tolerated^1^. Serological testing subsequently returned positive for *R. conorii* (IgM titer 1:64). Ampicillin/sulbactam was promptly discontinued, and the patient was maintained on targeted antibiotic monotherapy with doxycycline. Repeat echocardiography on day 10 demonstrated an LVEF of 39% with a global longitudinal strain (GLS) of −11.9% (Figure 8), confirming a significant recovery trend.

### Cardiac magnetic resonance imaging

CMR imaging was performed two days after hospital discharge to characterize myocardial tissue injury. The study confirmed the diagnosis of acute myocarditis, fulfilling the 2018 Updated Lake Louise Criteria. Functional assessment demonstrated a preserved LVEF of 56% and a right ventricular ejection fraction (RVEF) of 51%, with normal biventricular volumes and no regional wall motion abnormalities, consistent with rapid functional recovery.

**Figure 4.**
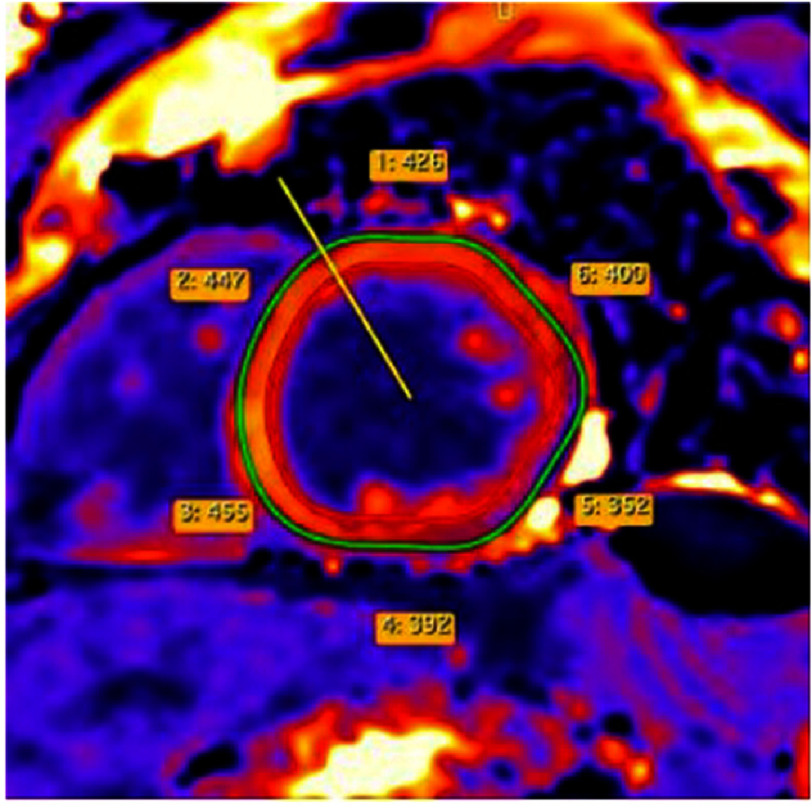
T1 mapping (basal, mid, and apical short-axis views) demonstrating values consistent with diffuse myocardial inflammation (normal range: 960–1020 ms, SIEMENS).

**Figure 5.**
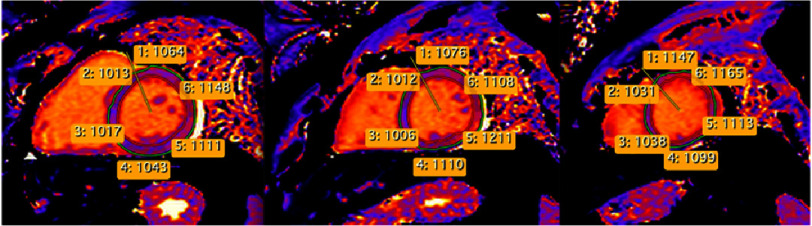
Post-contrast T1 mapping mid-short axis view.

**Figure 6.**
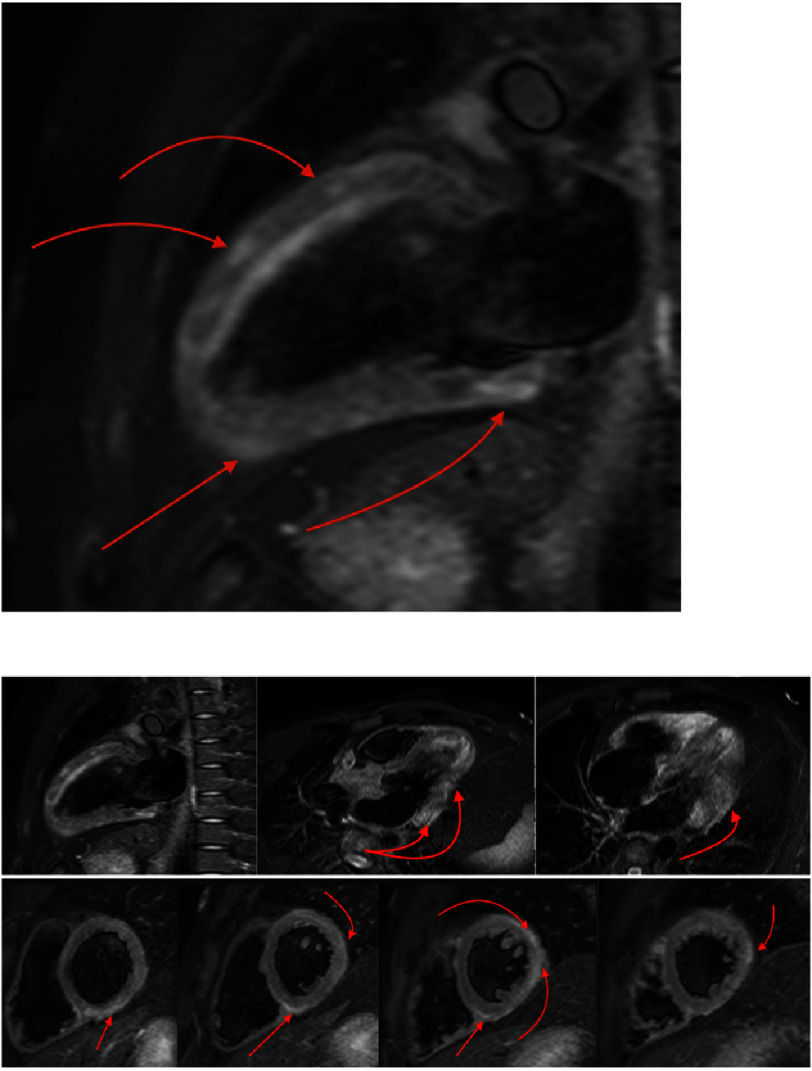
T2-weighted STIR images demonstrating myocardial edema and inflammation.

**Figure 7.**
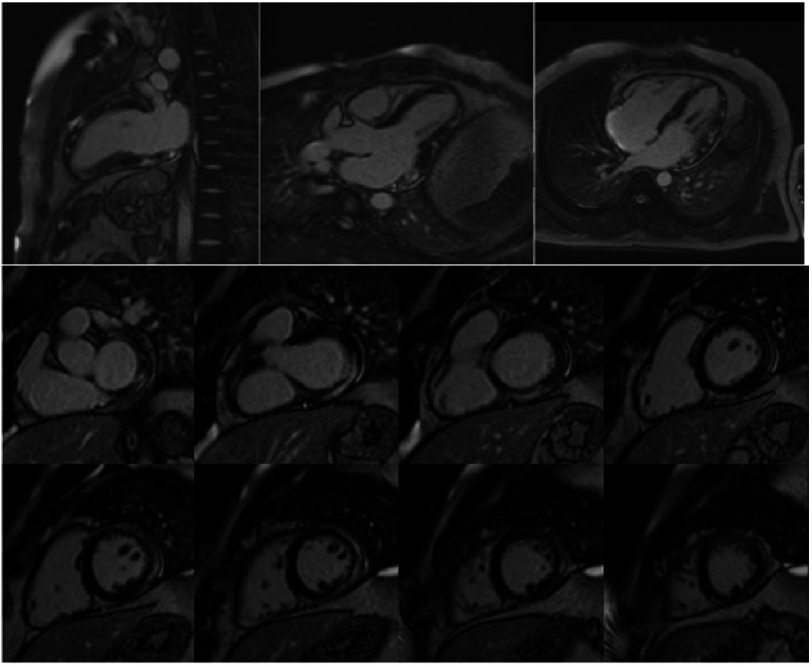
Late gadolinium enhancement (LGE) images demonstrating a non-ischemic pattern of myocardial enhancement.

Tissue characterization revealed extensive residual myocardial edema and inflammation. T2-weighted STIR sequences and T2 mapping demonstrated elevated values in the basal and mid inferior-inferolateral, anterior-anterolateral, and all apical segments (Figures 5–6). T1 mapping showed evidence of diffuse myocardial involvement consistent with acute myocarditis (Figure 4). LGE imaging displayed a characteristic non-ischemic pattern—epicardial and mid-myocardial layers—in the aforementioned segments, with additional linear mid-wall enhancement in the interventricular septum. The total LGE burden was estimated at 28% of LV mass; signs of resolving pericarditis were also identified (Figure 7).

**Figure 8.**
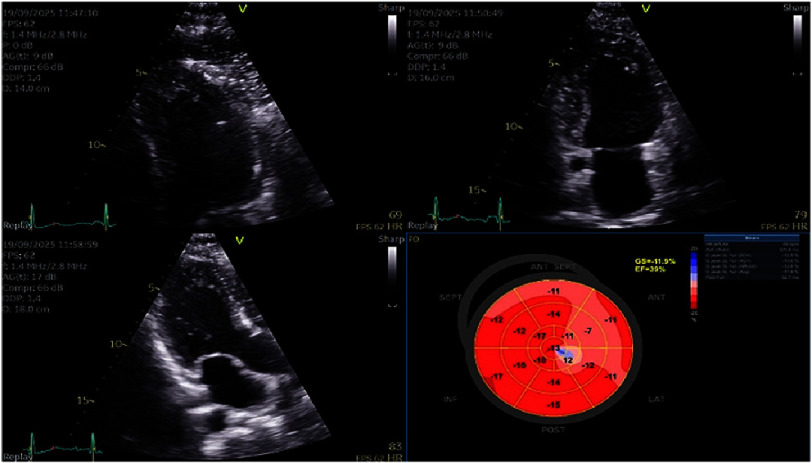
Echocardiographic assessment on day 10 of hospitalization demonstrating LVEF 39% and GLS −11.9%.

### Outcomes

The patient completed a 10-day hospitalization without complications. At discharge, echocardiography demonstrated improvement in LV systolic function, with an estimated LVEF of approximately 40% and a GLS of −11.9%. Heart failure therapy was continued at discharge, comprising ramipril, metoprolol, eplerenone, and empagliflozin.

### Follow-up

At 1-month follow-up, the patient remained asymptomatic and had resumed physical activity. Echocardiography demonstrated mild mitral regurgitation with preserved LV function (EF 55%) and normal RV function.

## Discussion

Rickettsioses are caused by bacteria of the genus *Rickettsia*—gram-negative obligate intracellular coccobacilli—transmitted by hematophagous arthropods, including ticks, fleas, and lice. They are serologically and genotypically classified into three major groups: the typhus group, the spotted fever group (SFG), and the transitional group.

The most prevalent rickettsial disease in Europe and the Mediterranean basin is Mediterranean spotted fever (MSF), caused by *R. conorii* and transmitted by the brown dog tick *Rhipicephalus sanguineus*, which is endemic to southern Europe, North Africa, and the Mediterranean region. Disease activity peaks during spring and summer when tick activity is greatest. Increasing ambient temperatures have been shown to enhance the aggressiveness of *R. sanguineus*, with potential implications for broader geographic spread and rising MSF incidence in future decades.

The highest reported annual incidence is in Portugal (9.8 cases per 100,000 population). In northern Greece, a seroepidemiological study detected *R. conorii* antibodies in 7.9% of the population, with higher prevalence among older individuals, men, and rural residents, suggesting frequent subclinical infection and probable underdiagnosis.

*R. conorii* targets vascular endothelial cells, causing microvasculitis and increased vascular permeability. Although MSF typically follows a benign course characterized by the classic triad of fever, rash, and inoculation eschar, complications occur in 1–20% of diagnosed cases, with mortality rates of 0–3%. Severe forms may involve the hepatic, renal, neurological, or pulmonary systems. Cardiac involvement is uncommon; pericarditis is the most frequent cardiac manifestation, while myocarditis and arrhythmias are exceptional.

While *R. conorii* is a recognized, albeit rare, cause of myopericarditis, the typical clinical course is predominantly mild, characterized by transient electrocardiographic changes and a favorable response to doxycycline. Historically, severe or fulminant presentations of MSF-associated myocarditis have been described almost exclusively in patients with significant preexisting comorbidities, such as hepatic insufficiency, congestive heart failure, or diabetes mellitus. Although catastrophic cardiac manifestations—including multifocal myocarditis, myocardial necrosis, and acute heart failure—have been well documented in related rickettsial infections such as Rocky Mountain spotted fever^2,3^, such presentations are exceedingly rare in *R. conorii* infection. To the best of our knowledge, the present case represents an exceptionally rare, if not the first, description of fulminant myocarditis complicated by acute hemodynamic collapse caused by *R. conorii* in a young, previously healthy, immunocompetent individual. The severity of this presentation—necessitating high-dose corticosteroids as rescue therapy alongside targeted antibiotic treatment—underscores the potential of this pathogen to trigger a life-threatening vasculitis and massive inflammatory cascade within the myocardium, even in otherwise healthy hosts.

## Conclusion

Myocarditis is an extremely rare complication of Mediterranean spotted fever and other *Rickettsia* infections, with only isolated case reports described in the literature. A high index of clinical suspicion is of paramount importance for timely diagnosis. Clinicians should correlate the patient’s symptoms with a history of insect bite in endemic regions, given the specific antibiotic therapy required and the fact that serological testing may yield false-negative results during the acute phase of infection.

## Funding

No funding was received.

## Conflicts of interest

The authors declare that they have no conflict of interest.

## Ethics statement

Patient’s informed consent for publication of the manuscript was obtained.
